# Factors associated with burnout among public hospital nurses in South Korea: A Job Demands–Resources perspective

**DOI:** 10.1371/journal.pone.0354729

**Published:** 2026-08-10

**Authors:** Ae Kyung Chang, Yoo Kyung Jin

**Affiliations:** 1 Professor, College of Nursing Science, Kyung Hee University, Seoul, South Korea; 2 College of Nursing Science, Kyung Hee University, Seoul, South Korea; Universiti Pertahanan Nasional Malaysia, MALAYSIA

## Abstract

Burnout among nurses in South Korean public hospitals is an important workforce issue in settings characterized by high job demands and resource constraints. This cross-sectional study examined burnout levels and factors associated with burnout among nurses working in five public hospitals in South Korea using pre-pandemic data collected in 2019. A total of 206 nurses completed validated questionnaires assessing burnout, workload, coworker support, mindfulness, and perceived nursing organizational culture. Demographic and work-related characteristics were also collected. Data were analyzed using descriptive statistics, t-tests, analysis of variance, Pearson’s correlation coefficients, and multiple linear regression. The mean burnout score was 3.10 out of 5. In the adjusted regression model, higher workload was associated with higher burnout (β = 0.53, *p* < 0.001), whereas higher mindfulness (β = −0.20, *p* < 0.001) and stronger perceived relationship-oriented nursing organizational culture (β = −0.19, *p* = 0.005) were associated with lower burnout. The model explained 58.0% of the variance in burnout. Coworker support and other organizational culture dimensions were not independently associated with burnout after adjustment for other factors. Therefore, burnout among nurses in public hospitals may be associated with both job demands and resources, particularly workload. These findings provide baseline evidence for future longitudinal and intervention research on burnout among public hospital nurses.

## Introduction

Public hospitals in South Korea, operated by national and local governments and other public-sector institutions, serve as critical safety-net providers within the national healthcare system. These hospitals deliver essential and emergency medical services to vulnerable populations, support infection control and disaster preparedness, and promote community health equity through education, public health initiatives, and workforce training [1]. Despite these roles, the capacity of the public hospital sector remains limited. In 2020, public hospital bed availability was 1.2 per 1,000 population, substantially below the Organization for Economic Co-operation and Development (OECD) average of 2.8, and in 2021, public hospitals accounted for only 9.5% of all hospital beds—the lowest proportion among OECD member nations [2]. This structural imbalance places sustained pressure on public hospitals, contributing to high workloads amid ongoing resource constraints.

These structural pressures are associated with heightened emotional, cognitive, and physical demands on nurses, who play a central role in sustaining essential services in public hospitals. Nurses are required to provide continuous patient monitoring, coordinate rapidly changing clinical situations, communicate with families, and meet administrative and regulatory expectations under strict time constraints. Chronic understaffing, limited financial resources, inadequate infrastructure, and rigid hierarchical cultures may further intensify these demands, increasing prolonged occupational stress and burnout [3]. Burnout—a multidimensional response to chronic work-related stress—has been consistently linked to poorer physical and psychological health, reduced work engagement, absenteeism, and higher turnover intentions among nurses, posing a substantial challenge to workforce sustainability [4].

Burnout is the inability to adequately cope with continuous job stress, manifested through emotional exhaustion, depersonalization, and a reduced sense of personal achievement [5]. As burnout among nurses intensifies, both physical health problems (e.g., cardiovascular symptoms, chronic pain, gastrointestinal disorders, and headaches) and emotional health problems (e.g., depression and insomnia) increase [6]. Consequently, job satisfaction decreases while turnover intention increases, exacerbating staffing shortages in clinical settings [7]. In particular, burnout increases the risk of patient safety incidents, such as falls and medication errors, leading to a decline in patient satisfaction and nursing care quality; thus, burnout reflects a critical issue that is directly linked to healthcare institution staffing and patient safety, extending beyond individual physical and emotional health problems [8, 9].

Within the Job Demands–Resources (JD-R) framework, burnout is conceptualized as emerging when job demands—such as high workload, role ambiguity, emotional labor, and shift-related stress—exceed available job resources [10]. While previous studies often explored burnout through attitudinal dimensions such as depersonalization [11, 12], the present study focuses on burnout as a state of physical, emotional, and mental exhaustion. This approach allows for a clearer distinction between burnout as an outcome and its relational and personal determinants. Specifically, conceptualizing burnout as a holistic state of exhaustion enables precise examination on how external environmental resources (e.g., organizational culture and coworker support) and internal regulatory resources (e.g., mindfulness) function to protect nurses’ core energy in the demanding context of public hospitals. In this study, workload is operationalized as a primary job demand, and coworker support and relationship-oriented organizational culture as job resources. Organizational context also plays an important role, as relational or innovative workplace cultures may buffer stress, whereas hierarchical or task-oriented cultures have been associated with higher burnout levels [13]. Furthermore, mindfulness is integrated into this framework as a personal resource functioning alongside environmental resources to mitigate burnout. Additionally, demographic and work-related characteristics, including younger age, shorter clinical experience, shift work, and limited social support, have been consistently associated with burnout across diverse healthcare settings [14, 15].

Despite growing global attention to nurse burnout, empirical research on nurse burnout in South Korean public hospitals remains limited, particularly in contexts characterized by structural constraints and elevated job demands. Furthermore, relatively few studies have examined burnout using data collected before the COVID-19 pandemic, although such baseline evidence is important for understanding longer-term patterns and for informing workforce support strategies. To address this gap, the present study examines burnout-associated factors, including sociodemographic characteristics, job demands (workload), job resources (organizational culture, coworker support), and personal resources (mindfulness) among nurses working in South Korean public hospitals. This research provides foundational evidence to inform organizational- and policy-level approaches to supporting the nursing workforce by identifying key correlations of burnout in the public healthcare sector.

## Materials and methods

### Research design and data collection

Data were collected between December 16 and 31, 2019, from nurses employed at five public hospitals in South Korea. This cross-sectional study examined the associations among workload, coworker support, mindfulness, organizational culture, and burnout among public hospital nurses and assessed the levels of burnout and factors associated with it. The study was designed and reported in accordance with the Strengthening the Reporting of Observational Studies in Epidemiology guidelines to promote comprehensive and transparent reporting. During the study design and data collection process, several procedural controls were implemented to reduce the potential for common method bias. Participants completed the questionnaire anonymously and were assured that their responses would remain confidential and would not affect their employment, performance evaluation, or workplace relationships. They were also informed that no responses would be considered right or wrong and were encouraged to respond honestly according to their own experiences. Additionally, the questionnaire was organized into separate sections for demographic characteristics, explanatory variables, and burnout to reduce evaluation apprehension and response patterning.

### Participants and settings

Nurses directly involved in patient care at five public hospitals in Seoul, South Korea, were included in this study. These hospitals were Seoul Medical Center, which is directly operated by the Seoul Metropolitan Government, and its affiliated municipal hospitals. These institutions included both tertiary- and secondary-level public hospitals, providing inpatient, outpatient, and community-based public healthcare services within the metropolitan public healthcare system. They served residents, including medically vulnerable populations, and functioned as regional public healthcare institutions. Detailed institutional characteristics are presented in aggregate rather than by individual hospitals to protect institutional confidentiality. To reflect variation in clinical work environments, this study recruited nurses from diverse clinical departments, particularly those with at least one year of clinical experience, to ensure sufficient exposure to their organizational culture and job demands. Participants received an explanation of the study, and written informed consent was obtained prior to participation. Questionnaires were distributed to consenting nurses and collected upon completion. Sample size estimation was conducted using G*Power version 3.1 (Faul et al., University of Düsseldorf, Düsseldorf, Germany; https://www.gpower.hhu.de/). Based on a previous study [16], an effect size of 0.15, a significance level of 0.05, and a statistical power of 0.95 indicated a minimum required sample size of 178. To account for a potential attrition rate of 20%, questionnaires were distributed to 220 nurses. A total of 206 nurses completed the survey, yielding a response rate of 93.6%.

### Measurements

#### Demographic characteristics.

Demographic characteristics included sex, age, marital status, and educational attainment. Job-related factors encompassed participants’ work unit, type of work, total career duration, and duration in the current unit.

#### Workload.

Workload was measured using the Copenhagen Psychosocial Questionnaire, originally developed by Pejtersen et al. [17] at the Statens Serum Institut in Denmark and adapted by Park [18] for use in hospital nursing settings. This 11-item instrument is divided into three subscales: quantitative demands (4 items), work pace (3 items), and emotional demands (4 items). Each item is rated on a five-point Likert scale ranging from 1 to 5, with higher scores indicating greater perceived workload. Cronbach’s α ranged from 0.82 to 0.87 during development and was 0.88 in the present study.

#### Coworker support.

Coworker support was measured using the Social Support Scale developed by Cutrona and Russell [19] and subsequently adapted by Yang [20]. This instrument, which assesses perceived social support, has five dimensions: attachment, social integration, opportunity for nurturance, reliable alliance, and guidance. Each item is rated on a five-point Likert scale ranging from 1 to 5, with higher scores indicating greater perceived support from coworkers. The mean score was used for the analysis. Cronbach’s α was 0.94 at the time of development and 0.92 in the present study.

#### Mindfulness.

Mindfulness was evaluated using a tool developed by Park [21] based on Vipassana meditation theory. This 20-item instrument consists of four subscales: present awareness (5 items), concentration (5 items), nonjudgmental acceptance (5 items), and decentered attention (5 items). Each item is rated on a 5-point Likert scale ranging from 1 to 5, with higher scores indicating greater mindfulness. Cronbach’s α was 0.88 in Park’s [21] study and 0.94 in the present study, indicating good reliability.

#### Nursing organizational culture.

Nursing organizational culture was assessed using the instrument developed by Kim et al. [22]. This instrument has four subscales: relationship-oriented culture, innovation-oriented culture, hierarchy-oriented culture, and task-oriented culture. Each item is rated on a five-point Likert scale ranging from 1 to 5, with higher scores for each culture type indicating that participants subjectively perceive their organizational culture as aligning more strongly with that type. The mean score for each subscale was used for the analysis. Nursing organizational culture refers to individual nurses’ subjective perceptions of organizational culture rather than objective organizational culture at the unit or hospital level. Reliability, as measured by Cronbach’s α, was 0.88 in the original study and 0.80 in the present study.

#### Burnout.

Burnout was measured using the Burnout Measure, originally developed by Malakh-Pines et al. [12] and subsequently adapted by Peek [11]. This instrument assesses burnout primarily as physical, emotional, and mental exhaustion. Each item is rated on a five-point Likert scale, with higher scores indicating greater burnout. In this study, the mean score represents nurses’ overall burnout. The Burnout Measure was selected because it captures overall burnout as an outcome variable while conceptually distinguishing burnout from its explanatory factors, including workload, coworker support, mindfulness, and perceived nursing organizational culture. Cronbach’s α was 0.86 in Peek’s [11] study and 0.90 in the present study.

### Data analysis

Data were analyzed using Statistical Package for the Social Sciences version 21.0 (IBM Corp., Armonk, NY, USA). Before analysis, the dataset was screened for missing values and outliers. Questionnaires with missing responses on key study variables were excluded, with no imputation performed. Therefore, complete-case analysis was conducted using data from participants providing complete responses. Participant demographic characteristics were initially summarized using frequencies, percentages, means, and standard deviations. Study variables—including workload, coworker support, mindfulness, perceived nursing organizational culture, and burnout—were described using means and standard deviations. Differences in burnout across participants’ general characteristics were examined using independent t-tests and one-way analysis of variance, after assessing normality and homogeneity of variance. Levene’s test was used for assessing homogeneity of variance, and Tukey’s honest significant difference test for post hoc comparisons. Associations among study variables were assessed using Pearson’s correlation coefficient. Finally, multiple linear regression analysis was conducted to identify factors associated with burnout. General characteristics showing significant differences in burnout in bivariate analyses were included as control variables. According to theoretical relevance and empirical associations with burnout, key explanatory variables included workload, coworker support, mindfulness, and perceived nursing organizational culture. Categorical variables were dummy-coded before entry into the regression model, with the reference categories specified in the regression table. Before interpreting the multiple linear regression results, assumptions including linearity, independence of errors, normality of residuals, homoscedasticity, absence of multicollinearity, and absence of influential observations were examined. The Durbin–Watson statistic was used for assessing independence of errors, and tolerance and variance inflation factor values for evaluating multicollinearity. Potential influential observations were assessed using standardized residuals and Cook’s distance. Regression results included unstandardized coefficients, standard errors, 95% confidence intervals for unstandardized coefficients, standardized coefficients, *p*-values, tolerance values, and variance inflation factor values. A *p*-value below .05 indicated statistical significance.

### Ethical considerations

This study was conducted in accordance with the Declaration of Helsinki. Ethical approval for data collection was obtained from the Seoul Medical Center Institutional Review Board (approval number: 2019-11-003-001), which reviewed the study’s objectives, methodology, confidentiality measures, data management procedures, information sheets, consent forms, and questionnaires. The written consent form explained the potential benefits and risks of participation, including measures to protect privacy and confidentiality, voluntary participation, the right to withdraw without disadvantage, and the exclusive use of data for research purposes. To maintain participant confidentiality, all questionnaires were completed anonymously.

## Results

### Participant characteristics and descriptive statistics

The participant cohort was predominantly female (n = 200, 97.1%), with a mean age of 33 years. Most participants were unmarried (n = 122, 59.2%), worked in general wards (n = 123, 59.7%), and were engaged in shift work (n = 170, 82.5%). Nearly half had less than 5 years of total clinical experience (n = 89, 43.2%), and the majority had less than 5 years of tenure in their current department (n = 183, 88.8%). Most participants held staff nurse positions (n = 194, 94.2%). The mean burnout score was 3.10 (SD = 0.55, range = 1–5), indicating a level above the midpoint (Table 1). Significant differences in burnout were observed based on age (*F* = 4.42, *p* = .013) and marital status (*t* = –3.38, *p* = .001). No significant differences were found for sex or educational level. Significant differences were also noted for shift work (*t* = 3.37, *p* = .001) and tenure in the current department (*F* = 3.31, *p* = .021), whereas total clinical experience and department type were not significantly associated with burnout (Table 2).

**Table 1:** Participant characteristics and descriptive statistics of study variables (*n* = 206).

Characteristics	Categories	*n* (%)	M ± SD
**Sex**	Men	6 (2.9%)	
	Women	200 (97.1%)	
**Age (years)**	<30	87 (42.2%)	
	30–39	73 (35.4%)	
	≥40	46 (22.4%)	
**Marital status**	Married	84 (40.8%)	
	Single	122 (59.2%)	
**Education level**	Diploma	46 (22.3%)	
	Bachelor	147 (71.4%)	
	Master’s or higher degree	13 (6.3%)	
**Work department**	General ward	123 (59.7%)	
	ICU	45 (21.8%)	
	Outpatient department	16 (7.8%)	
	Hemodialysis unit	22 (10.7%)	
**Shift work**	Yes	170 (82.5%)	
	No	36 (17.5%)	
**Years in nursing**	<5	89 (43.2%)	
	5–9	47 (22.8%)	
	10–14	30 (14.6%)	
	≥15	40 (19.4%)	
**Years in the current department**	<5	183 (88.8%)	
	5–9	13 (6.3%)	
	10–14	6 (2.9%)	
	≥15	4 (2.0%)	
**Job position**	Staff nurse	194 (94.2%)	
	Manager	12 (5.8%)	
**Burnout**			3.10 ± 0.55
**Workload**			2.96 ± 0.67
**Coworker support**			3.70 ± 0.55
**Mindfulness**			3.84 ± 0.69
**Nursing organizational culture**	Relationship-oriented culture		2.96 ± 0.75
	Hierarchy-oriented culture		3.35 ± 0.62
	Innovation-oriented culture		2.68 ± 0.62
	Task-oriented culture		2.44 ± 0.57

Unless otherwise stated, values are presented as n (%) or mean ± SDBurnout, workload, coworker support, mindfulness, and nursing organizational culture were measured using five-point Likert scales.

^†^Special units included the Intensive Care Unit and Hemodialysis unit.

**Table 2:** Differences in burnout according to participant characteristics (*n* = 206).

Characteristics	Categories	M ± SD	*t/F*(*p*) Tukey HSD
**Sex**	Men	2.89 ± 0.48	−0.94 (0.351)
	Women	3.10 ± 0.55	
**Age (years)**	<30^a^	3.19 ± 0.50	4.42 (.013) c < a
	30–39^b^	3.12 ± 0.57	
	≥40^c^	2.90 ± 0.56	
**Marital status**	Married	2.95 ± 0.52	−3.38 (.001)
	Single	3.20 ± 0.54	
**Education level**	Diploma	3.13 ± 0.59	0.09 (.911)
	Bachelor	3.09 ± 0.53	
	Master’s or higher degree	3.10 ± 0.65	
**Work department**	General ward	3.17 ± 0.51	1.73 (.162)
	Special units^†^	3.03 ± 0.59	
	Outpatient department	2.93 ± 0.64	
**Shift work**	Yes	3.16 ± 0.54	3.37 (.001)
	No	2.83 ± 0.48	
**Years in nursing**	<5	3.09 ± 0.53	2.39 (.070)
	5–9	3.15 ± 0.60	
	10–14	3.26 ± 0.62	
	≥15	2.93 ± 0.42	
**Years in the** **current department**	<5^a^	3.10 ± 0.55	3.31 (.021) c < b
	5–9^b^	3.38 ± 0.53	
	10–14^c^	2.63 ± 0.25	
	≥15^d^	2.74 ± 0.15	
**Job position**	Staff nurse	3.12 ± 0.54	2.23 (.045)
	Manager	2.78 ± 0.52	

a, b, c, d: Superscript letters indicate groups for Tukey's HSD post-hoc comparison test.

### Correlations between burnout and key variables

Burnout was positively correlated with workload (*r* = 0.678, *p* < .001) and negatively correlated with coworker support (*r* = -0.244, *p* < .001), mindfulness (*r* = -0.400, *p* < .001), relationship-oriented culture (r = -0.422, *p* < .001), innovation-oriented culture (*r* = -0.266, *p* < .001), and task-oriented culture (*r* = -0.165, *p* < .05) (Table 3). No significant correlation was observed between burnout and hierarchy-oriented culture (*r* = 0.021, *p* > .05).

**Table 3:** Correlation between workload, coworker support, mindfulness, nursing organizational culture, and burnout (n = 206).

Variables	1	2	3	4	5	6	7	8
**1. Burnout**	1							
**2. Workload**	.678^**^	1						
**3. Coworker support**	−.244^**^	−.167*	1					
**4. Mindfulness**	−.400^**^	−.297^**^	.245^**^	1				
**5. Relationship-oriented culture**	−.422^**^	−.296^**^	.387^**^	.153*	1			
**6. Hierarchy-oriented culture**	.021	.162*	.051	−.008	−.076	1		
**7. Innovation-oriented culture**	−.266^**^	−.160*	.174*	−.015	.635^**^	−.173*	1	
**8. Task-oriented culture**	−.165*	.057	.043	−.015	.265^**^	.179*	.386^**^	1

Values are Pearson’s correlation coefficients (r)

*p < .05, **p < .001 indicate statistical significance

### Predictors of burnout

The multiple regression model predicting burnout among public hospital nurses was significant (*F* = 19.69, *p* < .001) and explained 58.0% of the variance in burnout (adjusted R² = 0.58). Higher workload was associated with higher burnout (*β* = 0.53, *p* < .001), whereas higher mindfulness (*β* = –0.20, *p* < .001) and stronger relationship-oriented nursing organizational culture (*β* = –0.19, *p* = .005) were associated with lower burnout. Coworker support was not a significant predictor in the adjusted model (*β* = –0.01, *p* = .861), and hierarchy-, innovation-, and task-oriented culture subscales were also non-significant (all *p* > .05). After adjustment, demographic and work-related characteristics, including marital status, type of work shift, job position, age group, and years in the current unit—were not significantly associated with burnout (all *p* > .05) (Table 4).

**Table 4:** Effect of workload, coworker support, mindfulness, and nursing organizational culture on burnout (*n* = 206).

Variable		Unstandardized Coefficients		*Standardized Coefficients*	*t*	*p*	95% CI for *B*		Collinearity Statistics	
		B	SE	*β*			Lower	Upper	T	VIF
**Constant**		3.05	0.38		8.07	<.001	2.30	3.79		
**Workload**		0.44	0.04	0.53	10.15	<.001	0.35	0.52	0.75	1.33
**Coworker support**		−0.01	0.05	−0.01	−0.18	.861	−0.11	0.09	0.75	1.34
**Mindfulness**		−0.16	0.04	−0.20	−4.06	<.001	−0.24	−0.08	0.84	1.19
**Nursing organizational** **culture**	Relational	−0.14	0.05	−0.19	−2.82	.005	−0.23	−0.04	0.47	2.12
	Hierarchy	−0.05	0.04	−0.05	−1.05	.297	−0.13	0.04	0.84	1.19
	Innovation	−0.04	0.06	−0.04	−0.66	.507	−0.15	0.07	0.50	2.01
	Task	−0.09	0.05	−0.09	−1.61	.109	−0.19	0.02	0.69	1.46
Marital status (*Single)		0.11	0.06	0.10	1.81	.072	−0.01	0.24	0.63	1.59
Type of work shift (*Shift)		0.10	0.08	0.07	1.31	.193	−0.05	0.25	0.72	1.38
Job position (*Staff nurse)		−0.04	0.13	−0.02	−0.34	.736	−0.29	0.21	0.70	1.43
Age (* ≥ 40)	<30	0.08	0.09	0.07	0.92	.360	−0.09	0.25	0.34	2.97
	30–39	0.06	0.08	0.05	0.72	.471	−0.10	0.21	0.46	2.20
Years in the current unit (* ≥ 15)	<5	0.10	0.19	0.06	0.55	.581	−0.27	0.47	0.18	5.69
	5–9	0.25	0.21	0.11	1.20	.233	−0.16	0.67	0.23	4.31
	10–14	0.05	0.24	0.02	0.23	.822	−0.41	0.52	0.39	2.57
Durbin–Watson’s *d* = 1.95 (1.83 ≤ *d* ≤ 2.30); adj R² = 0.58; F = 19.69; *p* < 0.001										

Adjusted R², adjusted coefficient of determination; B, unstandardized coefficient; *F*, *F*-statistic; SE, standard error; t, t-statistic; *T*, tolerance; VIF, variance inflation factor; *β*, standardized coefficient

VIF: variance inflation factor

## Discussion

This study examined burnout among nurses working in South Korean public hospitals and explored potential associations with demographic, psychological, and organizational factors. Interpreted through the JD-R model, results suggest that burnout was associated with both job demands and resources. Workload, a core job demand, showed the strongest positive association with burnout, whereas mindfulness and perceived relationship-oriented nursing organizational culture, representing personal and organizational resources, were independently associated with lower burnout. This pattern aligns with the JD-R assumption that excessive job demands intensify exhaustion, whereas personal and organizational resources help nurses cope with or recover from work-related strain. Because the data were collected in 2019 prior to the COVID-19 pandemic, these findings provide a pre-pandemic benchmark under routine operating conditions. Such a benchmark may be useful for postpandemic workforce planning, particularly because structural constraints, including staffing capacity and workload intensity, may persist in the public hospital sector.

In bivariate analyses, several sociodemographic and job-related characteristics showed differences in burnout, but they were not independently associated with burnout after adjustment. Rather than interpreting that these characteristics are irrelevant to burnout, this finding indicates that their associations are partly explained by workload, mindfulness, perceived nursing organizational culture, or other psychosocial factors included in the multivariable model. From a workforce-management perspective, younger and early-career nurses, as well as shift-working nurses, may be more exposed to demanding work conditions or have less access to protective resources, thereby warranting further attention. Younger nurses often experience reality shock while transitioning into clinical practice and may be more vulnerable to high workload, insufficient adaptation support, limited autonomy, and unstable work environments [23]. Previous studies have also reported higher levels of stress and burnout among nurses younger than 35 years, whereas burnout levels tend to decrease with increasing age [24, 25]. Furthermore, shift work requires cautious interpretation. Although it was not independently associated with burnout in the adjusted model, it may disrupt sleep–wake rhythms, thereby limiting recovery opportunities and accumulating physical and psychological fatigue [26]. Beyond the emotional burden inherent in patient care, nurses often manage competing demands between paid work and family responsibilities [27]. Irregular days off and rotating schedules may further restrict opportunities for bonding with family and friends, thereby weakening interpersonal resources that could otherwise buffer work-related stress [28]. From the JD-R perspective, shift work may therefore contribute to burnout vulnerability by increasing physiological and emotional demands, reducing recovery time, and limiting access to social resources outside the workplace. For nurse managers, these findings underscore that structured transition support, fair workload allocation, and careful monitoring of fatigue and recovery opportunities are important among younger, early-career, and shift-working nurses.

In this study, burnout levels were substantial (mean score, 3.10), consistent with findings from other government-funded healthcare systems, where limited resources and increasing service demands may contribute to emotional fatigue [29]. As nurses’ burnout intensifies, both physical health problems (e.g., cardiovascular symptoms, chronic pain, gastrointestinal disorders, and headaches) and emotional health problems (e.g., depression and insomnia) increase [6]. Burnout also leads to decreased job satisfaction and increased turnover intention, thereby worsening staffing shortages in clinical settings [7]. In particular, it increases the risk of patient safety incidents, including falls and medication errors, ultimately declining patient satisfaction and nursing care quality; therefore, beyond its effect on individual physical and emotional well-being, burnout is a critical issue closely related to healthcare institution staffing and patient safety [8, 9]. Moreover, burnout may create a reinforcing cycle involving turnover intention, staffing gaps, and further workload intensification. Vacancies resulting from nurses leaving their jobs or exiting the workforce may increase the workload of remaining nurses. This work redistribution may also generate relational and moral strain, as nurses may feel that their unfinished work is transferred to colleagues, causing feelings of guilt and additional emotional burden [24]. Therefore, burnout among nurses should be understood within a broader framework that considers not only individual distress but also the job demands and organizational conditions that may sustain or intensify it.

Workload demonstrated the strongest independent association with burnout, and this finding can be interpreted through the JD-R model. Within this framework, excessive workload represents a core job demand consuming nurses’ physical, emotional, and cognitive resources, particularly when job resources are inadequate. In public hospitals, workload may reflect broader structural pressures, including staffing shortages, high patient-to-nurse ratios, administrative burden, limited infrastructure, and responsibility for providing essential services to vulnerable populations [3]. These circumstances may reduce recovery time, increasing vulnerability to physical, emotional, and mental exhaustion. Previous studies have similarly linked high workload and insufficient staffing with fatigue, emotional exhaustion, lower work engagement, and adverse workforce outcomes [30, 31]. From the JD-R perspective, the present findings suggest that workload should not be viewed merely as a quantitative task burden or a subjective feeling of being busy, but as a structural job demand that progressively diminishes nurses’ coping capacity when sustained without sufficient staffing, recovery time, fair workload distribution, and organizational support. Improving the work environment by increasing staffing capacity, clarifying work allocation, and reducing time spent on non-nursing or avoidable administrative tasks, may help nurses devote more time to direct patient care and mitigate workload-related strain [32]. Accordingly, rather than relying primarily on individual coping strategies, burnout prevention efforts should prioritize organizational and policy-level strategies aimed at improving objective working conditions—including adequate staffing, equitable workload distribution, reduced administrative burden, and leadership practices that recognize and respond to workload-related strain [33].

Within the JD-R model, mindfulness may be interpreted as a personal resource. Highly mindful nurses may be better able to recognize stressful experiences without immediate emotional reactivity, regulate negative emotions, and maintain attention in demanding clinical situations [34]. These capacities may help explain why mindfulness was independently negatively associated with burnout in this study. During clinical practice, nurses frequently encounter emotionally challenging situations, experiencing frustration, discouragement, anger, or emotional fatigue. Mindfulness may help nurses observe these emotional responses, reflect on them, and respond more flexibly rather than becoming overwhelmed by them. Mindfulness-based approaches may aid in coping with stress and burnout by enhancing emotional regulation, self-awareness, and resilience [34, 35]. However, given that the present study was cross-sectional and did not test a mindfulness intervention, mindfulness in this study may be interpreted as a potentially relevant personal resource rather than considering this finding as evidence that mindfulness education directly reduces burnout. Future intervention studies may consider flexible and accessible delivery formats, such as hybrid programs or app-based mindfulness training, to accommodate nursing work realities, including shift schedules, high work intensity, limited time, and difficulty participating in conventional face-to-face group education [36, 37].

While perceived relationship-oriented nursing organizational culture was independently associated with lower burnout after adjustment for other factors, perceived hierarchy-, innovation-, and task-oriented cultures were not. Unlike international studies, Korean nursing research commonly classifies nursing organizational culture into such four dimensions. In particular, relationship-oriented culture can be interpreted in relation to workplace social support, supportive work environments, and relational job resources, but it should not be equated with informal social support. Recent qualitative evidence suggests that, although workplace social support may include support from colleagues, immediate supervisors, and informal peer networks, nurses experience it mostly through spontaneous and immediate peer interactions when managing emotionally demanding work experiences [38]. While such informal support is valuable, it may depend heavily on individual initiative, trusted colleague availability, and nurses’ ability to seek and maintain supportive relationships. Conversely, perceived relationship-oriented culture reflects a more stable and organizationally embedded relational climate wherein shared norms, leadership practices, and work processes reinforce trust, open communication, mutual respect, and team-based support [22]. Therefore, while informal support plays an important role, it highlights the need for relational support to be more consistently embedded within organizational culture. From the JD-R perspective, perceived relationship-oriented culture may function as an organizational resource by reducing relational isolation, facilitating help-seeking behavior, supporting emotional recovery, and making relational support more reliably available in high-demand clinical environments. One of the present findings’ possible interpretations is that these relational resources may be especially relevant to burnout among public hospital nurses, whereas other cultural dimensions may be less directly connected to emotional recovery after adjustment for other factors. In public hospitals, where workload pressure and staffing constraints are substantial, informal peer support alone may insufficiently buffer structural job demands. Therefore, public hospitals should consider cultivating relationship-oriented organizational climates through leadership responsiveness, psychologically safe communication, team-based problem solving, structured opportunities for debriefing after difficult clinical events, and systems allowing nurses to seek support without stigma. These strategies should be considered potential organizational approaches rather than interventions whose effectiveness was established by the present cross-sectional study.

The nonsignificant independent association between coworker support and burnout in the adjusted model should also be interpreted cautiously. Workplace support reportedly can help nurses manage job pressures and reduce burnout risk; however, this is often examined using broader forms of support involving both colleagues and supervisors [39]. In the present study, only coworker support was measured, excluding supervisor support. This distinction is important because supervisor support may involve formal authority and access to organizational resources (e.g., staffing decisions, schedule adjustments, conflict resolution, and performance feedback), whereas coworker support primarily operates through peer-level emotional reassurance, shared understanding, and practical assistance during work. Therefore, rather than interpreting this finding as evidence that workplace support is unrelated to burnout, the present finding suggests that coworker support alone was not independently associated with burnout after accounting for workload, mindfulness, and perceived nursing organizational culture. Part of the protective meaning of coworker support may have also been captured by perceived relationship-oriented culture, which reflects a broader relational climate of trust, communication, mutual respect, and team-based support. Future studies should distinguish coworker support, supervisor support, and broader organizational support and examine their independent, combined, and potentially moderating associations with burnout.

Collectively, burnout among nurses in South Korean public hospitals should be viewed as a workforce and organizational issue shaped by the balance between job demands and available resources. In this study, workload was the strongest job demand associated with burnout, whereas mindfulness and perceived relationship-oriented nursing organizational culture appeared to represent personal and organizational resources associated with lower burnout levels. Therefore, to prevent burnout, public hospitals should enhance working conditions through adequate staffing, fair workload distribution, reduction of avoidable administrative and non-nursing tasks, and policies that support nurse recruitment and retention. Compensation structures, welfare benefits, and workforce incentives may also be relevant to sustain the public-sector nursing workforce, although this study did not directly measure these factors. Meanwhile, mindfulness and perceived relationship-oriented nursing organizational culture should be understood as complementary resources rather than substitutes for structural improvements. While mindfulness-related education may help strengthen nurses’ emotional regulation and coping capacity, a relationship-oriented organizational culture may support trust, open communication, mutual respect, psychological safety, and team-based problem-solving. Given the hierarchical characteristics and resource constraints potentially existing in nursing organizations within public hospital settings, leadership development and organizational culture strategies that foster relational support and responsive communication may be important for future management strategies and intervention studies.

This study has several limitations. First, the cross-sectional design and the use of self-reported data collected at a single time point preclude causal inference, potentially introducing reporting and common-method bias, despite implementing procedural controls such as anonymity, confidentiality, and questionnaire section separation. Second, because the sample was drawn from nurses in five public hospitals in Seoul, the findings may not be generalized to other public hospitals or private-sector settings with different staffing models, compensation structures, and organizational contexts. Third, unmeasured structural and socioeconomic factors—including unit-level staffing ratios, patient acuity, overtime workload, and leadership styles—may have influenced the observed associations. Notably, because the compensation systems of public hospitals in South Korea generally differ from those in the private sector, unmeasured financial dissatisfaction among public sector nurses may have confounded the relationship between workload and burnout. Finally, because the data were collected in 2019 and perceived nursing organizational culture was assessed at the individual rather than unit or hospital level, the findings cannot capture postpandemic changes or clarify contextual effects at higher organizational levels. Future longitudinal, repeated cross-sectional, and multilevel studies may address these limitations.

## Conclusion

This study identified workload, mindfulness, and perceived relationship-oriented nursing organizational culture as factors independently associated with burnout among nurses in South Korean public hospitals. Interpreted through the JD-R model, workload represents a core job demand, whereas mindfulness and perceived relationship-oriented nursing organizational culture may represent personal and organizational resources associated with lower burnout levels. Importantly, with workload showing the strongest association with burnout, public hospitals should prioritize structural improvements in objective working conditions to prevent burnout rather than relying primarily on individual coping strategies or cultural approaches. While mindfulness-related education and efforts to foster perceived relationship-oriented nursing organizational culture may support emotional regulation, communication, trust, and team-based support, they should complement rather than replace organizational and policy-level efforts to improve working conditions. Future longitudinal, multilevel, and intervention studies are needed to clarify causal pathways and evaluate whether improvements in job demands, personal resources, and organizational resources lead to sustained burnout reductions among public hospital nurses.
