# Supplementary material for: Factors associated with burnout among public hospital nurses in South Korea: A Job Demands–Resources perspective
Source: PLoS One. 2026 Aug 10;21(8):e0354729. doi: 10.1371/journal.pone.0354729 (PMC13456200; doi:10.1371/journal.pone.0354729)
Supplement: Supplementary file 1 [file pone.0354729.s002.docx]

**Reviewers’ Comments to the Authors:**

**Overall Evaluation**
This study uses a cross-sectional survey design and is based on the Job Demands–Resources model (JD-R model) to examine factors associated with nurse burnout in public hospitals in South Korea. The topic has clinical, managerial, and public health relevance, particularly in the context of resource constraints in public hospitals and growing concerns about nursing workforce stability. The manuscript is generally well structured. The ethics approval, informed consent procedures, and competing interest statement are clearly reported, and the overall statistical framework is acceptable.

: Thank you

However, several aspects of the manuscript require further revision, including the theoretical framework, wording related to the cross-sectional design, sample source and representativeness, measurement description, statistical reporting, interpretation of results, data availability statement, and academic writing. In particular, because this is a cross-sectional study, the temporal sequence and causal relationships among variables cannot be established. Therefore, the title and the manuscript as a whole should avoid terms such as “predictors,” “influencing factors,” “risk factors,” or “protective factors,” which may imply causal or predictive relationships. More appropriate wording would be “associated factors” or “factors associated with burnout.”
Overall, the manuscript has publication potential, but it requires major revision before further consideration.

**Response: We sincerely thank you for these valuable comments. The relevant terminology throughout the manuscript has been revised in accordance with your suggestions.**

**Reviewers’ Comments to the Authors: Reviewer 1**

**Major Comments
1. The research background, theoretical framework, and rationale for variable selection** **need to be strengthened**

The manuscript appropriately links nurse burnout to resource constraints in South Korean public hospitals and high nursing workload. However, the introduction does not provide sufficient explanation of the concept of burnout, the JD-R theoretical framework, or the rationale for selecting the study variables.
The authors should further clarify the core definition and theoretical meaning of burnout. They should also specify whether the burnout scale used in this study measures overall burnout or specific burnout dimensions. If the scale differs from commonly used three-dimensional burnout measures, the authors should explain the rationale for using this instrument and discuss the limitations when comparing the findings with previous studies.
In addition, the authors should explicitly clarify the theoretical position of each variable within the JD-R framework. For example, workload can be conceptualized as a job demand; co-worker support and relationship-oriented organizational culture can be conceptualized as job resources; mindfulness can be understood as a personal resource; and demographic and work-related characteristics can be treated as covariates. This would help establish a clearer logical connection among the research background, theoretical framework, variable selection, and statistical model.

**Response:**

**Addition of the concept of burnout**

Burnout is the inability to adequately cope with continuous job stress, manifested through emotional exhaustion, depersonalization, and a reduced sense of personal achievement [5]. As burnout among nurses intensifies, both physical health problems (e.g., cardiovascular symptoms, chronic pain, gastrointestinal disorders, and headaches) and emotional health problems (e.g., depression and insomnia) increase [6]. Consequently, job satisfaction decreases while turnover intention increases, exacerbating staffing shortages in clinical settings [7]. In particular, burnout increases the risk of patient safety incidents, such as falls and medication errors, leading to a decline in patient satisfaction and nursing care quality; thus, burnout reflects a critical issue that is directly linked to healthcare institution staffing and patient safety, extending beyond individual physical and emotional health problems [8,9].

**Explicit clarification of the theoretical position of each variable within the JD-R framework**

Within the Job Demands–Resources (JD-R) framework, burnout is conceptualized as emerging when job demands—such as high workload, role ambiguity, emotional labor, and shift-related stress—exceed available job resources [10]. While previous studies often explored burnout through attitudinal dimensions such as depersonalization [11,12], the present study focuses on burnout as a state of physical, emotional, and mental exhaustion. This approach allows for a clearer distinction between burnout as an outcome and its relational and personal determinants. Specifically, conceptualizing burnout as a holistic state of exhaustion enables precise examination on how external environmental resources (e.g., organizational culture and coworker support) and internal regulatory resources (e.g., mindfulness) function to protect nurses’ core energy in the demanding context of public hospitals. In this study, workload is operationalized as a primary job demand, and coworker support and relationship-oriented organizational culture as job resources. Organizational context also plays an important role, as relational or innovative workplace cultures may buffer stress, whereas hierarchical or task-oriented cultures have been associated with higher burnout levels [13]. Furthermore, mindfulness is integrated into this framework as a personal resource functioning alongside environmental resources to mitigate burnout. Additionally, demographic and work-related characteristics, including younger age, shorter clinical experience, shift work, and limited social support, have been consistently associated with burnout across diverse healthcare settings [14,15].

**Burnout measurement**

While previous studies often explored burnout through attitudinal dimensions such as depersonalization [11,12], the present study focuses on burnout as a state of physical, emotional, and mental exhaustion.

**2. The title, research objectives, and wording throughout the manuscript should avoid causal or predictive language**
The current title uses the term “Predictors.” However, because this study has a cross-sectional design, it cannot establish temporal order among variables. Therefore, wording that implies prediction or causality is not appropriate. For example, in the abstract, introduction, discussion, and conclusion, terms such as “predict,” “effect,” “influence,” and “determinants” should be systematically checked and replaced with terms such as “associated with,” “related to,” or “correlates of,” which are more consistent with the level of evidence provided by a cross-sectional study.

**Response: Yes, we have revised the relevant terminology throughout the manuscript according to your feedback. Thank you.**

**3. More information is needed regarding the sample source, sampling method, and representativeness**
The authors should provide more detailed information about the sample source and sampling process. It is currently unclear how the five public hospitals were selected, whether convenience sampling was used, and whether these hospitals can represent nurses working in public hospitals in South Korea.
The authors should clarify the criteria for selecting the hospitals, their geographic distribution, hospital size or type, inclusion and exclusion criteria for nurses, whether temporary nurses or nurses in non-clinical positions were included, and whether differences between responders and non-responders were assessed.
Because the sample was drawn from only five public hospitals and consisted mainly of female nurses and staff nurses, the authors should explicitly acknowledge the homogeneity of the sample and its implications for generalizability in the limitations section. If the sample is not nationally representative, the conclusions should not be generalized to all public hospital nurses in South Korea or to the broader nursing population.

**Response: Thank you for your review. We have revised the wording in line with your feedback.**

Nurses directly involved in patient care at five public hospitals in Seoul, South Korea, were included in this study. These hospitals were Seoul Medical Center, which is directly operated by the Seoul Metropolitan Government, and its affiliated municipal hospitals. These institutions included both tertiary- and secondary-level public hospitals, providing inpatient, outpatient, and community-based public healthcare services within the metropolitan public healthcare system. They served residents, including medically vulnerable populations, and functioned as regional public healthcare institutions. Detailed institutional characteristics are presented in aggregate rather than by individual hospitals to protect institutional confidentiality. To reflect variation in clinical work environments, this study recruited nurses from diverse clinical departments, particularly those with at least one year of clinical experience, to ensure sufficient exposure to their organizational culture and job demands.

**4. The measurement instruments should be described in greater detail**
The authors should provide more complete information about each scale, including the number of items, dimensional structure, scoring method, score range, whether total scores or mean scores were used, the meaning of higher scores, existing reliability and validity evidence, and whether the instruments are appropriate for Korean nursing populations.
For the burnout scale, the authors should clarify whether it measures overall burnout or specific dimensions of burnout and explain the rationale for selecting this instrument. If the instrument differs from widely used tools such as the Maslach Burnout Inventory or the Copenhagen Burnout Inventory, the authors should discuss the limitations of comparing the results with previous studies.
For nursing organizational culture, the authors should clearly state that the variable reflects individuals’ subjective perceptions of organizational culture rather than organizational culture at the unit or hospital level. Accordingly, the conclusions should avoid interpreting individual-level perceptions as objective organizational-level cultural characteristics.

**Response: The content has been added and modified according to your feedback for each measurement.**

Measurements

Demographic characteristics: Demographic characteristics included sex, age, marital status, and educational attainment. Job-related factors encompassed participants’ work unit, type of work, total career duration, and duration in the current unit.

Workload: Workload was measured using the Copenhagen Psychosocial Questionnaire, originally developed by Pejtersen et al. [17] at the Statens Serum Institut in Denmark and adapted by Park [18] for use in hospital nursing settings. This 11-item instrument is divided into three subscales: quantitative demands (4 items), work pace (3 items), and emotional demands (4 items). Each item is rated on a five-point Likert scale ranging from 1 to 5, with higher scores indicating greater perceived workload. Cronbach’s α ranged from 0.82 to 0.87 during development and was 0.88 in the present study.

Coworker support: Coworker support was measured using the Social Support Scale developed by Cutrona and Russell [19] and subsequently adapted by Yang [20]. This instrument, which assesses perceived social support, has five dimensions: attachment, social integration, opportunity for nurturance, reliable alliance, and guidance. Each item is rated on a five-point Likert scale ranging from 1 to 5, with higher scores indicating greater perceived support from coworkers. The mean score was used for the analysis. Cronbach’s α was 0.94 at the time of development and 0.92 in the present study.

Mindfulness: Mindfulness was evaluated using a tool developed by Park (2006) based on Vipassana meditation theory. This 20-item instrument consists of four subscales: present awareness (5 items), concentration (5 items), nonjudgmental acceptance (5 items), and decentered attention (5 items). Each item is rated on a 5-point Likert scale ranging from 1 to 5, with higher scores indicating greater mindfulness. Cronbach’s α was 0.88 in Park’s (2006) study and 0.94 in the present study, indicating good reliability.

Nursing organizational culture: Nursing organizational culture was assessed using the instrument developed by Kim et al. [22]. This instrument has four subscales: relationship-oriented culture, innovation-oriented culture, hierarchy-oriented culture, and task-oriented culture. Each item is rated on a five-point Likert scale ranging from 1 to 5, with higher scores for each culture type indicating that participants subjectively perceive their organizational culture as aligning more strongly with that type. The mean score for each subscale was used for the analysis. Nursing organizational culture refers to individual nurses’ subjective perceptions of organizational culture rather than objective organizational culture at the unit or hospital level. Reliability, as measured by Cronbach’s α, was 0.88 in the original study and 0.80 in the present study.

Burnout: Burnout was measured using the Burnout Measure, originally developed by Malakh-Pines et al. [12] and subsequently adapted by Peek [11]. This instrument assesses burnout primarily as physical, emotional, and mental exhaustion. Each item is rated on a five-point Likert scale, with higher scores indicating greater burnout. In this study, the mean score represents nurses’ overall burnout. The Burnout Measure was selected because it captures overall burnout as an outcome variable while conceptually distinguishing burnout from its explanatory factors, including workload, coworker support, mindfulness, and perceived nursing organizational culture. Cronbach’s α was 0.86 in Peek’s [11] study and 0.90 in the present study.

**5. The statistical analysis should be reported more transparently**
Although the manuscript uses standard statistical methods, including descriptive statistics, t-tests, ANOVA, correlation analysis, and multiple linear regression, additional details are needed to improve reproducibility.
The authors should clarify whether there were missing data and how they were handled; whether the assumptions of t-tests, ANOVA, Pearson correlation, and linear regression were examined; how categorical variables were coded in the regression model; what reference categories were used; the rationale for including variables in the multivariable regression model; and whether outliers, influential observations, residual distributions, linearity, homoscedasticity, and multicollinearity were assessed.
In addition, the regression results should include 95% confidence intervals for the unstandardized regression coefficients. Reporting only p values and standardized coefficients is insufficient for evaluating the precision and uncertainty of the estimates.

**Response: Thank you. Questionnaires with missing responses were excluded from the analysis, and the analysis statements have been revised and rewritten as suggested..**

Data Analysis

Data were analyzed using Statistical Package for the Social Sciences version 21.0 (IBM Corp., Armonk, NY, USA). Before analysis, the dataset was screened for missing values and outliers. Questionnaires with missing responses on key study variables were excluded, with no imputation performed. Therefore, complete-case analysis was conducted using data from participants providing complete responses. Participant demographic characteristics were initially summarized using frequencies, percentages, means, and standard deviations. Study variables—including workload, coworker support, mindfulness, perceived nursing organizational culture, and burnout—were described using means and standard deviations. Differences in burnout across participants’ general characteristics were examined using independent t-tests and one-way analysis of variance, after assessing normality and homogeneity of variance. Levene’s test was used for assessing homogeneity of variance, and Tukey’s honest significant difference test for post hoc comparisons. Associations among study variables were assessed using Pearson’s correlation coefficient. Finally, multiple linear regression analysis was conducted to identify factors associated with burnout. General characteristics showing significant differences in burnout in bivariate analyses were included as control variables. According to theoretical relevance and empirical associations with burnout, key explanatory variables included workload, coworker support, mindfulness, and perceived nursing organizational culture. Categorical variables were dummy-coded before entry into the regression model, with the reference categories specified in the regression table. Before interpreting the multiple linear regression results, assumptions including linearity, independence of errors, normality of residuals, homoscedasticity, absence of multicollinearity, and absence of influential observations were examined. The Durbin–Watson statistic was used for assessing independence of errors, and tolerance and variance inflation factor values for evaluating multicollinearity. Potential influential observations were assessed using standardized residuals and Cook’s distance. Regression results included unstandardized coefficients, standard errors, 95% confidence intervals for unstandardized coefficients, standardized coefficients, *p*-values, tolerance values, and variance inflation factor values. A *p*-value below .05 indicated statistical significance.

**6. Common method bias should be assessed or discussed**
All major variables in this study were measured using self-report questionnaires at the same time point, which may introduce common method bias. The authors should state whether any procedural controls were implemented during study design or data collection, such as anonymous responses, reducing evaluation apprehension, or separating different variable sections in the questionnaire.
If possible, the authors may add statistical tests for common method bias, such as Harman’s single-factor test or other more appropriate methods. Even if additional statistical testing is not feasible, common method bias should be explicitly discussed in the limitations section, particularly given the relatively high explained variance of the model.

**Response: Yes, thank you. Such content has been added to the paragraph describing the study limitations.**

This study has several limitations. First, the cross-sectional design and the use of self-reported data collected at a single time point preclude causal inference, potentially introducing reporting and common-method bias, despite implementing procedural controls such as anonymity, confidentiality, and questionnaire section separation.

**7. The interpretation of regression results should be more cautious**
The authors should more clearly distinguish among univariate analyses, correlation analyses, and multivariable regression analyses. Some demographic or work-related variables may be significant in univariate analyses but non-significant in the multivariable regression model. This does not necessarily mean that these variables are irrelevant. Rather, their associations with burnout may be partly explained by workload, mindfulness, organizational culture, or other variables.
Similarly, if co-worker support or some dimensions of organizational culture are significant in correlation analyses but non-significant in the multivariable model, the authors should not simply conclude that these factors are unrelated to burnout. A more appropriate interpretation would be that, after controlling for other variables, these factors did not show independent associations with burnout, possibly due to shared variance with other resource-related variables.

**Response: In line with your suggestions, we have completely revised the relevant content in the Discussion section.**

**8. The discussion should provide stronger theoretical interpretation and engagement with the literature**
The discussion section needs further development. The authors should reduce simple repetition of the results and provide deeper theoretical interpretation based on the JD-R model. For example, they should explain why workload, as a job demand, was associated with higher burnout, and why mindfulness and relationship-oriented organizational culture, as personal or organizational resources, were associated with lower burnout.
The authors should also compare their findings more systematically with both Korean and international nursing studies, explaining consistencies and discrepancies and discussing possible reasons. In particular, the authors should further discuss the institutional context of public hospitals, such as resource constraints, staffing pressures, public health responsibilities, and administrative burden, and how these factors may contribute to nurse burnout.
The practical implications should also be stated more cautiously. The authors may discuss workload management, mindfulness-related training, relationship-oriented organizational culture, shift optimization, and leadership support as potentially relevant directions. However, these should be described as possible management strategies or potential targets for future intervention research, rather than as interventions proven effective by the present study.

**Response: Yes, the relevant content in the Discussion section has been revised accordingly.**

**Minor Comments**
1. The abstract should be further streamlined and should clearly present the study design, sample, major variables, statistical methods, and key findings. The conclusion should avoid causal or intervention-oriented language.

**Response: Thank you. Corrections have been made according to your suggestions.**

2. Terminology should be used consistently throughout the manuscript. For example, “co-worker support” and “coworker support” should not be used interchangeably; similarly, terms such as “workload,” “worker load,” and “nursing workload” should be standardized.

**Response: Thank you for your feedback. The text has been revised to standardize the terminology.**

3. The names of organizational culture dimensions should be standardized, such as relationship-oriented culture, hierarchy-oriented culture, innovation-oriented culture, and task-oriented culture.

**Response: We appreciate your comment. Likewise, such terminologies have been revised for standardization.**

4. The tables should be carefully checked, including variable names, footnotes, abbreviation definitions, reference categories, p-value formatting, and statistical symbols. The regression table should include 95% confidence intervals.

**Response: Thank you. The tables have been revised according to your suggestions.**

5. The format for reporting p values should be standardized, for example, using p < .001 rather than p = 0.000. The authors should avoid interpreting results based solely on statistical significance.

**Response: Thank you. Corrections have been made accordingly.**

6. Abbreviations should be spelled out at first use, such as JD-R, VIF, and IRB.

**Response: Thank you. These abbreviations are now defined at their first appearance in the main text.**

7. The reference format should be further standardized. The authors are also encouraged to include more recent studies from the past three to five years on nurse burnout, nursing organizational culture, mindfulness, and the JD-R model.

**Response: Thank you for this helpful comment. The reference format has been standardized according to the journal’s preferred style. We also reviewed recent literature on nurse burnout, nursing organizational culture, mindfulness, and the Job Demands–Resources model. Given that some of the measurement instruments used in this study are original or Korean-adapted tools, we retained the original development and Korean adaptation studies as essential methodological references. However, recent empirical and review studies have been added, where appropriate, to strengthen the Introduction and Discussion sections.**

8. The English writing requires further polishing, especially in the abstract and discussion. The authors should check long sentences, grammatical completeness, subject–verb agreement, terminology consistency, and the use of causal verbs.

**Response: Thank you. Corrections have been made accordingly.**

**Reviewers’ Comments to the Authors: Reviewer 2**

This manuscript presents original research based on a cross-sectional survey conducted among nurses working in public hospitals in South Korea. The study aims to measure burnout levels and identify their key predictors, including workload, co-worker support, mindfulness, and nursing organizational culture. The manuscript is well-written, clearly structured, and possesses a solid methodological foundation. The theoretical framework (Job Demands–Resources model) is appropriate and consistently applied throughout the paper. The use of pre-pandemic data represents a meaningful contribution to the literature, as such benchmarks are valuable for post-pandemic workforce planning. The statistical approach is generally sound, with an adequately powered sample, a high response rate, and a regression model that explains a substantial proportion of the variance in burnout. I recommend minor revisions before acceptance

**Response: Thank you very much.**

**1.** Manuscript states that data were collected from five public hospitals in South Korea, there is no descriptive information about these institutions. Key characteristics such as geographic region, hospital level or tier (tertiary, secondary), size (number of beds), and the population each hospital serves is needed.

**Response: We appreciate your review. The content has been revised as suggested.**

Nurses directly involved in patient care at five public hospitals in Seoul, South Korea, were included in this study. These hospitals were Seoul Medical Center, which is directly operated by the Seoul Metropolitan Government, and its affiliated municipal hospitals. These institutions included both tertiary- and secondary-level public hospitals, providing inpatient, outpatient, and community-based public healthcare services within the metropolitan public healthcare system. They served residents, including medically vulnerable populations, and functioned as regional public healthcare institutions. Detailed institutional characteristics are presented in aggregate rather than by individual hospitals to protect institutional confidentiality. To reflect variation in clinical work environments, this study recruited nurses from diverse clinical departments, particularly those with at least one year of clinical experience, to ensure sufficient exposure to their organizational culture and job demands.

**2.** Special units comprised only the intensive care unit and the artificial kidney unit. Please, describe participants from each units.

**Response: The relevant content has been added and modified according to your feedback in Table 1.**

**3.** Financial conditions are well-established correlates of job satisfaction and burnout in the nursing literature, particularly in public-sector settings where salaries may differ substantially from private hospitals. The current study does not collect or discuss salary-related variables. While the authors may not have had access to this information, the omission should be explicitly acknowledged in the limitations section.

**Response: Thank you for your review. The relevant content has been revised accordingly**.

In public hospitals, workload may reflect broader structural pressures, including staffing shortages, high patient-to-nurse ratios, administrative burden, limited infrastructure, and responsibility for providing essential services to vulnerable populations [3]. These circumstances may reduce recovery time, increasing vulnerability to physical, emotional, and mental exhaustion. Previous studies have similarly linked high workload and insufficient staffing with fatigue, emotional exhaustion, lower work engagement, and adverse workforce outcomes [30,31].
**4.** The descriptive data reveal that a substantial proportion of participants were younger nurses with limited clinical experience: 42.2% were under 30 years of age, 43.2% had fewer than five years of total nursing experience, and 88.8% had fewer than five years in their current department. If public hospitals systematically employ less experienced nurses, this has important implications for workload distribution, mentorship needs, and burnout risk that go beyond individual-level factors.

**Response: Thank you for this important observation. We have revised the Discussion section to address the high proportion of younger and less experienced nurses in the sample.**

Younger nurses often experience reality shock while transitioning into clinical practice and may be more vulnerable to high workload, insufficient adaptation support, limited autonomy, and unstable work environments [23]. Previous studies have also reported higher levels of stress and burnout among nurses younger than 35 years, whereas burnout levels tend to decrease with increasing age [24,25]. Furthermore, shift work requires cautious interpretation. Although it was not independently associated with burnout in the adjusted model, it may disrupt sleep–wake rhythms, thereby limiting recovery opportunities and accumulating physical and psychological fatigue [26]. Beyond the emotional burden inherent in patient care, nurses often manage competing demands between paid work and family responsibilities [27]. Irregular days off and rotating schedules may further restrict opportunities for bonding with family and friends, thereby weakening interpersonal resources that could otherwise buffer work-related stress [28]. From the JD-R perspective, shift work may therefore contribute to burnout vulnerability by increasing physiological and emotional demands, reducing recovery time, and limiting access to social resources outside the workplace. For nurse managers, these findings underscore that structured transition support, fair workload allocation, and careful monitoring of fatigue and recovery opportunities are important among younger, early-career, and shift-working nurses.

**5.** Consider adding a figure illustrating the key predictors of burnout.

**Response: Thank you for this helpful suggestion. We agree that the key findings may be interpreted well through a visual summary. After considering this suggestion, we decided not to add a separate figure because the revised regression table already details the direction, magnitude, and precision of the associations, including 95% confidence intervals for the unstandardized coefficients. To avoid redundancy, we instead strengthened the regression table and clarified the key findings in the Results section.**

**6.** The manuscript provides a compelling description of the structural conditions prevailing in South Korean public hospitals, painting a challenging picture of chronic resource constraints, high patient-to-nurse ratios, staffing limitations, and insufficient infrastructure. The introduction clearly situates these systemic pressures as the broader context within which burnout emerges, and the data confirm that workload — arguably the most direct expression of these structural deficits — is by far the strongest predictor of burnout.. Despite this, the recommendations offered in the discussion lean predominantly toward individually-oriented or cultural interventions, particularly the promotion of mindfulness practices and the fostering of relationship-oriented organizational culture. The authors are encouraged to more explicitly acknowledge this tension. Improving objective working conditions may represent far more powerful levers for burnout prevention than individual coping strategies.

**Response: Thank you for this insightful comment. We agree that the Discussion section should more explicitly acknowledge the tension between the structural context described in the Introduction and recommendations focused on individual or cultural interventions. We have revised the Discussion section, emphasizing that workload was the strongest factor associated with burnout in the adjusted model and that burnout prevention strategies should prioritize improving objective working conditions, including staffing capacity, workload distribution, patient-to-nurse ratios, and administrative burden. We also clarified that mindfulness-based strategies and efforts to strengthen perceived relationship-oriented nursing organizational culture should be considered complementary approaches rather than substitutes for organizational and policy-level interventions aimed at reducing excessive workload.**
