# Supplementary material for: Factors associated with burnout among public hospital nurses in South Korea: A Job Demands–Resources perspective
Source: PLoS One. 2026 Aug 10;21(8):e0354729. doi: 10.1371/journal.pone.0354729 (PMC13456200; doi:10.1371/journal.pone.0354729)
Supplement: Peer review history

# Peer review: Decision Letter 0

**DOI:** 10.1371/journal.pone.0354729.r001

**Authors:** Rogis Baker

29 Apr 2026

PONE-D-26-11716

Predictors of nurse burnout in public hospitals: A cross-sectional survey

PLOS One

Dear Dr. Kyung,

Thank you for submitting your manuscript to PLOS ONE. After careful consideration, we feel that it has merit but does not fully meet PLOS ONE’s publication criteria as it currently stands. Therefore, we invite you to submit a revised version of the manuscript that addresses the points raised during the review process.

Please submit your revised manuscript by Jun 13 2026 11:59PM. If you will need more time than this to complete your revisions, please reply to this message or contact the journal office at plosone@plos.org. When you're ready to submit your revision, log on to https://www.editorialmanager.com/pone/ and select the 'Submissions Needing Revision' folder to locate your manuscript file.

Please include the following items when submitting your revised manuscript:

- A letter that responds to each point raised by the academic editor and reviewer(s). You should upload this letter as a separate file labeled 'Response to Reviewers'.
- A marked-up copy of your manuscript that highlights changes made to the original version. You should upload this as a separate file labeled 'Revised Manuscript with Track Changes'.
- An unmarked version of your revised paper without tracked changes. You should upload this as a separate file labeled 'Manuscript'.

If you would like to make changes to your financial disclosure, please include your updated statement in your cover letter. Guidelines for resubmitting your figure files are available below the reviewer comments at the end of this letter.

If applicable, we recommend that you deposit your laboratory protocols in protocols.io to enhance the reproducibility of your results. Protocols.io assigns your protocol its own identifier (DOI) so that it can be cited independently in the future. For instructions see: https://journals.plos.org/plosone/s/submission-guidelines#loc-laboratory-protocols. Additionally, PLOS ONE offers an option for publishing peer-reviewed Lab Protocol articles, which describe protocols hosted on protocols.io. Read more information on sharing protocols at https://plos.org/protocols?utm_medium=editorial-email&utm_source=authorletters&utm_campaign=protocols.

As the corresponding author, your ORCID iD is verified in the submission system and will appear in the published article. PLOS supports the use of ORCID, and we encourage all coauthors to register for an ORCID iD and use it as well. Please encourage your coauthors to verify their ORCID iD within the submission system before final acceptance, as unverified ORCID iDs will not appear in the published article. *Only*  the individual author can complete the verification step; PLOS staff *cannot*  verify ORCID iDs on behalf of authors.

We look forward to receiving your revised manuscript.

Kind regards,

Rogis Baker, Ph.D

Academic Editor

PLOS One

**Journal Requirements:**

When submitting your revision, we need you to address these additional requirements.

1. Please ensure that your manuscript meets PLOS ONE's style requirements, including those for file naming. The PLOS ONE style templates can be found at

https://journals.plos.org/plosone/s/file?id=wjVg/PLOSOne_formatting_sample_main_body.pdf and

https://journals.plos.org/plosone/s/file?id=ba62/PLOSOne_formatting_sample_title_authors_affiliations.pdf

2. In the online submission form you indicate that your data is not available for proprietary reasons and have provided a contact point for accessing this data. Please note that your current contact point is a co-author on this manuscript. According to our Data Policy, the contact point must not be an author on the manuscript and must be an institutional contact, ideally not an individual. Please revise your data statement to a non-author institutional point of contact, such as a data access or ethics committee, and send this to us via return email. Please also include contact information for the third party organization, and please include the full citation of where the data can be found.

3. If the reviewer comments include a recommendation to cite specific previously published works, please review and evaluate these publications to determine whether they are relevant and should be cited. There is no requirement to cite these works unless the editor has indicated otherwise.

[Note: HTML markup is below. Please do not edit.]

Reviewers' comments:

Reviewer's Responses to Questions

**Comments to the Author**

1. Is the manuscript technically sound, and do the data support the conclusions?

The manuscript must describe a technically sound piece of scientific research with data that supports the conclusions. Experiments must have been conducted rigorously, with appropriate controls, replication, and sample sizes. The conclusions must be drawn appropriately based on the data presented.

Reviewer #1: Yes

Reviewer #2: Yes

**********

2. Has the statistical analysis been performed appropriately and rigorously?

Reviewer #1: Yes

Reviewer #2: Yes

**********

3. Have the authors made all data underlying the findings in their manuscript fully available?

The PLOS Data policy requires authors to make all data underlying the findings described in their manuscript fully available without restriction, with rare exception (please refer to the Data Availability Statement in the manuscript PDF file). The data should be provided as part of the manuscript or its supporting information, or deposited to a public repository. For example, in addition to summary statistics, the data points behind means, medians and variance measures should be available. If there are restrictions on publicly sharing data—e.g. participant privacy or use of data from a third party—those must be specified.

Reviewer #1: Yes

Reviewer #2: Yes

**********

4. Is the manuscript presented in an intelligible fashion and written in standard English?

PLOS ONE does not copyedit accepted manuscripts, so the language in submitted articles must be clear, correct, and unambiguous. Any typographical or grammatical errors should be corrected at revision, so please note any specific errors here.

Reviewer #1: Yes

Reviewer #2: Yes

**********

5. Review Comments to the Author

Please use the space provided to explain your answers to the questions above. You may also include additional comments for the author, including concerns about dual publication, research ethics, or publication ethics. (Please upload your review as an attachment if it exceeds 20,000 characters)

Reviewer #1: Review Comments

Manuscript ID: PONE-D-26-11716

Manuscript Title: Predictors of nurse burnout in public hospitals: A cross-sectional survey

1. Overall Evaluation

This study uses a cross-sectional survey design and is based on the Job Demands–Resources model (JD-R model) to examine factors associated with nurse burnout in public hospitals in South Korea. The topic has clinical, managerial, and public health relevance, particularly in the context of resource constraints in public hospitals and growing concerns about nursing workforce stability. The manuscript is generally well structured. The ethics approval, informed consent procedures, and competing interest statement are clearly reported, and the overall statistical framework is acceptable.

However, several aspects of the manuscript require further revision, including the theoretical framework, wording related to the cross-sectional design, sample source and representativeness, measurement description, statistical reporting, interpretation of results, data availability statement, and academic writing. In particular, because this is a cross-sectional study, the temporal sequence and causal relationships among variables cannot be established. Therefore, the title and the manuscript as a whole should avoid terms such as “predictors,” “influencing factors,” “risk factors,” or “protective factors,” which may imply causal or predictive relationships. More appropriate wording would be “associated factors” or “factors associated with burnout.”

Overall, the manuscript has publication potential, but it requires major revision before further consideration.

2. Major Comments

1. The research background, theoretical framework, and rationale for variable selection need to be strengthened

The manuscript appropriately links nurse burnout to resource constraints in South Korean public hospitals and high nursing workload. However, the introduction does not provide sufficient explanation of the concept of burnout, the JD-R theoretical framework, or the rationale for selecting the study variables.

The authors should further clarify the core definition and theoretical meaning of burnout. They should also specify whether the burnout scale used in this study measures overall burnout or specific burnout dimensions. If the scale differs from commonly used three-dimensional burnout measures, the authors should explain the rationale for using this instrument and discuss the limitations when comparing the findings with previous studies.

In addition, the authors should explicitly clarify the theoretical position of each variable within the JD-R framework. For example, workload can be conceptualized as a job demand; co-worker support and relationship-oriented organizational culture can be conceptualized as job resources; mindfulness can be understood as a personal resource; and demographic and work-related characteristics can be treated as covariates. This would help establish a clearer logical connection among the research background, theoretical framework, variable selection, and statistical model.

2. The title, research objectives, and wording throughout the manuscript should avoid causal or predictive language

The current title uses the term “Predictors.” However, because this study has a cross-sectional design, it cannot establish temporal order among variables. Therefore, wording that implies prediction or causality is not appropriate. For example, in the abstract, introduction, discussion, and conclusion, terms such as “predict,” “effect,” “influence,” and “determinants” should be systematically checked and replaced with terms such as “associated with,” “related to,” or “correlates of,” which are more consistent with the level of evidence provided by a cross-sectional study.

3. More information is needed regarding the sample source, sampling method, and representativeness

The authors should provide more detailed information about the sample source and sampling process. It is currently unclear how the five public hospitals were selected, whether convenience sampling was used, and whether these hospitals can represent nurses working in public hospitals in South Korea.

The authors should clarify the criteria for selecting the hospitals, their geographic distribution, hospital size or type, inclusion and exclusion criteria for nurses, whether temporary nurses or nurses in non-clinical positions were included, and whether differences between responders and non-responders were assessed.

Because the sample was drawn from only five public hospitals and consisted mainly of female nurses and staff nurses, the authors should explicitly acknowledge the homogeneity of the sample and its implications for generalizability in the limitations section. If the sample is not nationally representative, the conclusions should not be generalized to all public hospital nurses in South Korea or to the broader nursing population.

4. The measurement instruments should be described in greater detail

The authors should provide more complete information about each scale, including the number of items, dimensional structure, scoring method, score range, whether total scores or mean scores were used, the meaning of higher scores, existing reliability and validity evidence, and whether the instruments are appropriate for Korean nursing populations.

For the burnout scale, the authors should clarify whether it measures overall burnout or specific dimensions of burnout and explain the rationale for selecting this instrument. If the instrument differs from widely used tools such as the Maslach Burnout Inventory or the Copenhagen Burnout Inventory, the authors should discuss the limitations of comparing the results with previous studies.

For nursing organizational culture, the authors should clearly state that the variable reflects individuals’ subjective perceptions of organizational culture rather than organizational culture at the unit or hospital level. Accordingly, the conclusions should avoid interpreting individual-level perceptions as objective organizational-level cultural characteristics.

5. The statistical analysis should be reported more transparently

Although the manuscript uses standard statistical methods, including descriptive statistics, t-tests, ANOVA, correlation analysis, and multiple linear regression, additional details are needed to improve reproducibility.

The authors should clarify whether there were missing data and how they were handled; whether the assumptions of t-tests, ANOVA, Pearson correlation, and linear regression were examined; how categorical variables were coded in the regression model; what reference categories were used; the rationale for including variables in the multivariable regression model; and whether outliers, influential observations, residual distributions, linearity, homoscedasticity, and multicollinearity were assessed.

In addition, the regression results should include 95% confidence intervals for the unstandardized regression coefficients. Reporting only p values and standardized coefficients is insufficient for evaluating the precision and uncertainty of the estimates.

6. Common method bias should be assessed or discussed

All major variables in this study were measured using self-report questionnaires at the same time point, which may introduce common method bias. The authors should state whether any procedural controls were implemented during study design or data collection, such as anonymous responses, reducing evaluation apprehension, or separating different variable sections in the questionnaire.

If possible, the authors may add statistical tests for common method bias, such as Harman’s single-factor test or other more appropriate methods. Even if additional statistical testing is not feasible, common method bias should be explicitly discussed in the limitations section, particularly given the relatively high explained variance of the model.

7. The interpretation of regression results should be more cautious

The authors should more clearly distinguish among univariate analyses, correlation analyses, and multivariable regression analyses. Some demographic or work-related variables may be significant in univariate analyses but non-significant in the multivariable regression model. This does not necessarily mean that these variables are irrelevant. Rather, their associations with burnout may be partly explained by workload, mindfulness, organizational culture, or other variables.

Similarly, if co-worker support or some dimensions of organizational culture are significant in correlation analyses but non-significant in the multivariable model, the authors should not simply conclude that these factors are unrelated to burnout. A more appropriate interpretation would be that, after controlling for other variables, these factors did not show independent associations with burnout, possibly due to shared variance with other resource-related variables.

8. The discussion should provide stronger theoretical interpretation and engagement with the literature

The discussion section needs further development. The authors should reduce simple repetition of the results and provide deeper theoretical interpretation based on the JD-R model. For example, they should explain why workload, as a job demand, was associated with higher burnout, and why mindfulness and relationship-oriented organizational culture, as personal or organizational resources, were associated with lower burnout.

The authors should also compare their findings more systematically with both Korean and international nursing studies, explaining consistencies and discrepancies and discussing possible reasons. In particular, the authors should further discuss the institutional context of public hospitals, such as resource constraints, staffing pressures, public health responsibilities, and administrative burden, and how these factors may contribute to nurse burnout.

The practical implications should also be stated more cautiously. The authors may discuss workload management, mindfulness-related training, relationship-oriented organizational culture, shift optimization, and leadership support as potentially relevant directions. However, these should be described as possible management strategies or potential targets for future intervention research, rather than as interventions proven effective by the present study.

3. Minor Comments

1. The abstract should be further streamlined and should clearly present the study design, sample, major variables, statistical methods, and key findings. The conclusion should avoid causal or intervention-oriented language.

2. Terminology should be used consistently throughout the manuscript. For example, “co-worker support” and “coworker support” should not be used interchangeably; similarly, terms such as “workload,” “worker load,” and “nursing workload” should be standardized.

3. The names of organizational culture dimensions should be standardized, such as relationship-oriented culture, hierarchy-oriented culture, innovation-oriented culture, and task-oriented culture.

4. The tables should be carefully checked, including variable names, footnotes, abbreviation definitions, reference categories, p-value formatting, and statistical symbols. The regression table should include 95% confidence intervals.

5. The format for reporting p values should be standardized, for example, using p < .001 rather than p = 0.000. The authors should avoid interpreting results based solely on statistical significance.

6. Abbreviations should be spelled out at first use, such as JD-R, VIF, and IRB.

7. The reference format should be further standardized. The authors are also encouraged to include more recent studies from the past three to five years on nurse burnout, nursing organizational culture, mindfulness, and the JD-R model.

8. The English writing requires further polishing, especially in the abstract and discussion. The authors should check long sentences, grammatical completeness, subject–verb agreement, terminology consistency, and the use of causal verbs.

Reviewer #2: This manuscript presents original research based on a cross-sectional survey conducted among nurses working in public hospitals in South Korea. The study aims to measure burnout levels and identify their key predictors, including workload, co-worker support, mindfulness, and nursing organizational culture. The manuscript is well-written, clearly structured, and possesses a solid methodological foundation. The theoretical framework (Job Demands–Resources model) is appropriate and consistently applied throughout the paper. The use of pre-pandemic data represents a meaningful contribution to the literature, as such benchmarks are valuable for post-pandemic workforce planning. The statistical approach is generally sound, with an adequately powered sample, a high response rate, and a regression model that explains a substantial proportion of the variance in burnout. I recommend minor revisions before acceptance:

- The manuscript states that data were collected from five public hospitals in South Korea, there is no descriptive information about these institutions. Key characteristics such as geographic region, hospital level or tier (tertiary, secondary), size (number of beds), and the population each hospital serves is needed.

- Special units comprised only the intensive care unit and the artificial kidney unit. Please, describe participants from each units.

- Financial conditions are well-established correlates of job satisfaction and burnout in the nursing literature, particularly in public-sector settings where salaries may differ substantially from private hospitals. The current study does not collect or discuss salary-related variables. While the authors may not have had access to this information, the omission should be explicitly acknowledged in the limitations section.

- The descriptive data reveal that a substantial proportion of participants were younger nurses with limited clinical experience: 42.2% were under 30 years of age, 43.2% had fewer than five years of total nursing experience, and 88.8% had fewer than five years in their current department. If public hospitals systematically employ less experienced nurses, this has important implications for workload distribution, mentorship needs, and burnout risk that go beyond individual-level factors.

- Consider adding a figure illustrating the key predictors of burnout.

- The manuscript provides a compelling description of the structural conditions prevailing in South Korean public hospitals, painting a challenging picture of chronic resource constraints, high patient-to-nurse ratios, staffing limitations, and insufficient infrastructure. The introduction clearly situates these systemic pressures as the broader context within which burnout emerges, and the data confirm that workload — arguably the most direct expression of these structural deficits — is by far the strongest predictor of burnout.

Despite this, the recommendations offered in the discussion lean predominantly toward individually-oriented or cultural interventions, particularly the promotion of mindfulness practices and the fostering of relationship-oriented organizational culture. The authors are encouraged to more explicitly acknowledge this tension. Improving objective working conditions may represent far more powerful levers for burnout prevention than individual coping strategies.

**********

6. PLOS authors have the option to publish the peer review history of their article (what does this mean?). If published, this will include your full peer review and any attached files.

If you choose “no”, your identity will remain anonymous but your review may still be made public.

**Do you want your identity to be public for this peer review?**  For information about this choice, including consent withdrawal, please see our Privacy Policy.

Reviewer #1: No

Reviewer #2: **Yes:**  Xacobe Abel Fernández García

**********

[NOTE: If reviewer comments were submitted as an attachment file, they will be attached to this email and accessible via the submission site. Please log into your account, locate the manuscript record, and check for the action link "View Attachments". If this link does not appear, there are no attachment files.]

To ensure your figures meet our technical requirements, please review our figure guidelines: https://journals.plos.org/plosone/s/figures

You may also use PLOS’s free figure tool, NAAS, to help you prepare publication quality figures: https://journals.plos.org/plosone/s/figures#loc-tools-for-figure-preparation.

NAAS will assess whether your figures meet our technical requirements by comparing each figure against our figure specifications.

# Author comment: Author response to Decision Letter 1

**DOI:** 10.1371/journal.pone.0354729.r002

11 Jun 2026

Reviewers’ Comments to the Authors:

Overall Evaluation

This study uses a cross-sectional survey design and is based on the Job Demands–Resources model (JD-R model) to examine factors associated with nurse burnout in public hospitals in South Korea. The topic has clinical, managerial, and public health relevance, particularly in the context of resource constraints in public hospitals and growing concerns about nursing workforce stability. The manuscript is generally well structured. The ethics approval, informed consent procedures, and competing interest statement are clearly reported, and the overall statistical framework is acceptable.

: Thank you

However, several aspects of the manuscript require further revision, including the theoretical framework, wording related to the cross-sectional design, sample source and representativeness, measurement description, statistical reporting, interpretation of results, data availability statement, and academic writing. In particular, because this is a cross-sectional study, the temporal sequence and causal relationships among variables cannot be established. Therefore, the title and the manuscript as a whole should avoid terms such as “predictors,” “influencing factors,” “risk factors,” or “protective factors,” which may imply causal or predictive relationships. More appropriate wording would be “associated factors” or “factors associated with burnout.”

Overall, the manuscript has publication potential, but it requires major revision before further consideration.

Response: We sincerely thank you for these valuable comments. The relevant terminology throughout the manuscript has been revised in accordance with your suggestions.

Reviewers’ Comments to the Authors: Reviewer 1

Major Comments

1. The research background, theoretical framework, and rationale for variable selection need to be strengthened

The manuscript appropriately links nurse burnout to resource constraints in South Korean public hospitals and high nursing workload. However, the introduction does not provide sufficient explanation of the concept of burnout, the JD-R theoretical framework, or the rationale for selecting the study variables.

The authors should further clarify the core definition and theoretical meaning of burnout. They should also specify whether the burnout scale used in this study measures overall burnout or specific burnout dimensions. If the scale differs from commonly used three-dimensional burnout measures, the authors should explain the rationale for using this instrument and discuss the limitations when comparing the findings with previous studies.

In addition, the authors should explicitly clarify the theoretical position of each variable within the JD-R framework. For example, workload can be conceptualized as a job demand; co-worker support and relationship-oriented organizational culture can be conceptualized as job resources; mindfulness can be understood as a personal resource; and demographic and work-related characteristics can be treated as covariates. This would help establish a clearer logical connection among the research background, theoretical framework, variable selection, and statistical model.

Response:

Addition of the concept of burnout

Burnout is the inability to adequately cope with continuous job stress, manifested through emotional exhaustion, depersonalization, and a reduced sense of personal achievement [5]. As burnout among nurses intensifies, both physical health problems (e.g., cardiovascular symptoms, chronic pain, gastrointestinal disorders, and headaches) and emotional health problems (e.g., depression and insomnia) increase [6]. Consequently, job satisfaction decreases while turnover intention increases, exacerbating staffing shortages in clinical settings [7]. In particular, burnout increases the risk of patient safety incidents, such as falls and medication errors, leading to a decline in patient satisfaction and nursing care quality; thus, burnout reflects a critical issue that is directly linked to healthcare institution staffing and patient safety, extending beyond individual physical and emotional health problems [8,9].

Explicit clarification of the theoretical position of each variable within the JD-R framework

Within the Job Demands–Resources (JD-R) framework, burnout is conceptualized as emerging when job demands—such as high workload, role ambiguity, emotional labor, and shift-related stress—exceed available job resources [10]. While previous studies often explored burnout through attitudinal dimensions such as depersonalization [11,12], the present study focuses on burnout as a state of physical, emotional, and mental exhaustion. This approach allows for a clearer distinction between burnout as an outcome and its relational and personal determinants. Specifically, conceptualizing burnout as a holistic state of exhaustion enables precise examination on how external environmental resources (e.g., organizational culture and coworker support) and internal regulatory resources (e.g., mindfulness) function to protect nurses’ core energy in the demanding context of public hospitals. In this study, workload is operationalized as a primary job demand, and coworker support and relationship-oriented organizational culture as job resources. Organizational context also plays an important role, as relational or innovative workplace cultures may buffer stress, whereas hierarchical or task-oriented cultures have been associated with higher burnout levels [13]. Furthermore, mindfulness is integrated into this framework as a personal resource functioning alongside environmental resources to mitigate burnout. Additionally, demographic and work-related characteristics, including younger age, shorter clinical experience, shift work, and limited social support, have been consistently associated with burnout across diverse healthcare settings [14,15].

Burnout measurement

While previous studies often explored burnout through attitudinal dimensions such as depersonalization [11,12], the present study focuses on burnout as a state of physical, emotional, and mental exhaustion.

2. The title, research objectives, and wording throughout the manuscript should avoid causal or predictive language

The current title uses the term “Predictors.” However, because this study has a cross-sectional design, it cannot establish temporal order among variables. Therefore, wording that implies prediction or causality is not appropriate. For example, in the abstract, introduction, discussion, and conclusion, terms such as “predict,” “effect,” “influence,” and “determinants” should be systematically checked and replaced with terms such as “associated with,” “related to,” or “correlates of,” which are more consistent with the level of evidence provided by a cross-sectional study.

Response: Yes, we have revised the relevant terminology throughout the manuscript according to your feedback. Thank you.

3. More information is needed regarding the sample source, sampling method, and representativeness

The authors should provide more detailed information about the sample source and sampling process. It is currently unclear how the five public hospitals were selected, whether convenience sampling was used, and whether these hospitals can represent nurses working in public hospitals in South Korea.

The authors should clarify the criteria for selecting the hospitals, their geographic distribution, hospital size or type, inclusion and exclusion criteria for nurses, whether temporary nurses or nurses in non-clinical positions were included, and whether differences between responders and non-responders were assessed.

Because the sample was drawn from only five public hospitals and consisted mainly of female nurses and staff nurses, the authors should explicitly acknowledge the homogeneity of the sample and its implications for generalizability in the limitations section. If the sample is not nationally representative, the conclusions should not be generalized to all public hospital nurses in South Korea or to the broader nursing population.

Response: Thank you for your review. We have revised the wording in line with your feedback.

Nurses directly involved in patient care at five public hospitals in Seoul, South Korea, were included in this study. These hospitals were Seoul Medical Center, which is directly operated by the Seoul Metropolitan Government, and its affiliated municipal hospitals. These institutions included both tertiary- and secondary-level public hospitals, providing inpatient, outpatient, and community-based public healthcare services within the metropolitan public healthcare system. They served residents, including medically vulnerable populations, and functioned as regional public healthcare institutions. Detailed institutional characteristics are presented in aggregate rather than by individual hospitals to protect institutional confidentiality. To reflect variation in clinical work environments, this study recruited nurses from diverse clinical departments, particularly those with at least one year of clinical experience, to ensure sufficient exposure to their organizational culture and job demands.

4. The measurement instruments should be described in greater detail

The authors should provide more complete information about each scale, including the number of items, dimensional structure, scoring method, score range, whether total scores or mean scores were used, the meaning of higher scores, existing reliability and validity evidence, and whether the instruments are appropriate for Korean nursing populations.

For the burnout scale, the authors should clarify whether it measures overall burnout or specific dimensions of burnout and explain the rationale for selecting this instrument. If the instrument differs from widely used tools such as the Maslach Burnout Inventory or the Copenhagen Burnout Inventory, the authors should discuss the limitations of comparing the results with previous studies.

For nursing organizational culture, the authors should clearly state that the variable reflects individuals’ subjective perceptions of organizational culture rather than organizational culture at the unit or hospital level. Accordingly, the conclusions should avoid interpreting individual-level perceptions as objective organizational-level cultural characteristics.

Response: The content has been added and modified according to your feedback for each measurement.

Measurements

Demographic characteristics: Demographic characteristics included sex, age, marital status, and educational attainment. Job-related factors encompassed participants’ work unit, type of work, total career duration, and duration in the current unit.

Workload: Workload was measured using the Copenhagen Psychosocial Questionnaire, originally developed by Pejtersen et al. [17] at the Statens Serum Institut in Denmark and adapted by Park [18] for use in hospital nursing settings. This 11-item instrument is divided into three subscales: quantitative demands (4 items), work pace (3 items), and emotional demands (4 items). Each item is rated on a five-point Likert scale ranging from 1 to 5, with higher scores indicating greater perceived workload. Cronbach’s α ranged from 0.82 to 0.87 during development and was 0.88 in the present study.

Coworker support: Coworker support was measured using the Social Support Scale developed by Cutrona and Russell [19] and subsequently adapted by Yang [20]. This instrument, which assesses perceived social support, has five dimensions: attachment, social integration, opportunity for nurturance, reliable alliance, and guidance. Each item is rated on a five-point Likert scale ranging from 1 to 5, with higher scores indicating greater perceived support from coworkers. The mean score was used for the analysis. Cronbach’s α was 0.94 at the time of development and 0.92 in the present study.

Mindfulness: Mindfulness was evaluated using a tool developed by Park (2006) based on Vipassana meditation theory. This 20-item instrument consists of four subscales: present awareness (5 items), concentration (5 items), nonjudgmental acceptance (5 items), and decentered attention (5 items). Each item is rated on a 5-point Likert scale ranging from 1 to 5, with higher scores indicating greater mindfulness. Cronbach’s α was 0.88 in Park’s (2006) study and 0.94 in the present study, indicating good reliability.

Nursing organizational culture: Nursing organizational culture was assessed using the instrument developed by Kim et al. [22]. This instrument has four subscales: relationship-oriented culture, innovation-oriented culture, hierarchy-oriented culture, and task-oriented culture. Each item is rated on a five-point Likert scale ranging from 1 to 5, with higher scores for each culture type indicating that participants subjectively perceive their organizational culture as aligning more strongly with that type. The mean score for each subscale was used for the analysis. Nursing organizational culture refers to individual nurses’ subjective perceptions of organizational culture rather than objective organizational culture at the unit or hospital level. Reliability, as measured by Cronbach’s α, was 0.88 in the original study and 0.80 in the present study.

Burnout: Burnout was measured using the Burnout Measure, originally developed by Malakh-Pines et al. [12] and subsequently adapted by Peek [11]. This instrument assesses burnout primarily as physical, emotional, and mental exhaustion. Each item is rated on a five-point Likert scale, with higher scores indicating greater burnout. In this study, the mean score represents nurses’ overall burnout. The Burnout Measure was selected because it captures overall burnout as an outcome variable while conceptually distinguishing burnout from its explanatory factors, including workload, coworker support, mindfulness, and perceived nursing organizational culture. Cronbach’s α was 0.86 in Peek’s [11] study and 0.90 in the present study.

5. The statistical analysis should be reported more transparently

Although the manuscript uses standard statistical methods, including descriptive statistics, t-tests, ANOVA, correlation analysis, and multiple linear regression, additional details are needed to improve reproducibility.

The authors should clarify whether there were missing data and how they were handled; whether the assumptions of t-tests, ANOVA, Pearson correlation, and linear regression were examined; how categorical variables were coded in the regression model; what reference categories were used; the rationale for including variables in the multivariable regression model; and whether outliers, influential observations, residual distributions, linearity, homoscedasticity, and multicollinearity were assessed.

In addition, the regression results should include 95% confidence intervals for the unstandardized regression coefficients. Reporting only p values and standardized coefficients is insufficient for evaluating the precision and uncertainty of the estimates.

Response: Thank you. Questionnaires with missing responses were excluded from the analysis, and the analysis statements have been revised and rewritten as suggested..

Data Analysis

Data were analyzed using Statistical Package for the Social Sciences version 21.0 (IBM Corp., Armonk, NY, USA). Before analysis, the dataset was screened for missing values and outliers. Questionnaires with missing responses on key study variables were excluded, with no imputation performed. Therefore, complete-case analysis was conducted using data from participants providing complete responses. Participant demographic characteristics were initially summarized using frequencies, percentages, means, and standard deviations. Study variables—including workload, coworker support, mindfulness, perceived nursing organizational culture, and burnout—were described using means and standard deviations. Differences in burnout across participants’ general characteristics were examined using independent t-tests and one-way analysis of variance, after assessing normality and homogeneity of variance. Levene’s test was used for assessing homogeneity of variance, and Tukey’s honest significant difference test for post hoc comparisons. Associations among study

Attachment Submitted filename: Response to reviewer.docx

# Peer review: Decision Letter 1

**DOI:** 10.1371/journal.pone.0354729.r003

**Authors:** Rogis Baker

13 Jul 2026

Factors associated with burnout among public hospital nurses in South Korea: A Job Demands–Resources perspective

PONE-D-26-11716R1

Dear Kyung,

We’re pleased to inform you that your manuscript has been judged scientifically suitable for publication and will be formally accepted for publication once it meets all outstanding technical requirements.

Within one week, you’ll receive an e-mail detailing the required amendments. When these have been addressed, you’ll receive a formal acceptance letter and your manuscript will be scheduled for publication.

An invoice will be generated when your article is formally accepted. Please note, if your institution has a publishing partnership with PLOS and your article meets the relevant criteria, all or part of your publication costs will be covered. Please make sure your user information is up-to-date by logging into Editorial Manager at Editorial Manager® and clicking the ‘Update My Information' link at the top of the page. For questions related to billing, please contact billing support.

If your institution or institutions have a press office, please notify them about your upcoming paper to help maximize its impact. If they’ll be preparing press materials, please inform our press team as soon as possible -- no later than 48 hours after receiving the formal acceptance. Your manuscript will remain under strict press embargo until 2 pm Eastern Time on the date of publication. For more information, please contact onepress@plos.org.

Kind regards,

Rogis Baker, Ph.D

Academic Editor

PLOS One

Additional Editor Comments (optional):

Reviewers' comments:

Reviewer's Responses to Questions

**Comments to the Author**

1. If the authors have adequately addressed your comments raised in a previous round of review and you feel that this manuscript is now acceptable for publication, you may indicate that here to bypass the “Comments to the Author” section, enter your conflict of interest statement in the “Confidential to Editor” section, and submit your "Accept" recommendation.

Reviewer #2: All comments have been addressed

**********

2. Is the manuscript technically sound, and do the data support the conclusions?

The manuscript must describe a technically sound piece of scientific research with data that supports the conclusions. Experiments must have been conducted rigorously, with appropriate controls, replication, and sample sizes. The conclusions must be drawn appropriately based on the data presented.

Reviewer #2: Yes

**********

3. Has the statistical analysis been performed appropriately and rigorously?

Reviewer #2: Yes

**********

4. Have the authors made all data underlying the findings in their manuscript fully available?

The PLOS Data policy requires authors to make all data underlying the findings described in their manuscript fully available without restriction, with rare exception (please refer to the Data Availability Statement in the manuscript PDF file). The data should be provided as part of the manuscript or its supporting information, or deposited to a public repository. For example, in addition to summary statistics, the data points behind means, medians and variance measures should be available. If there are restrictions on publicly sharing data—e.g. participant privacy or use of data from a third party—those must be specified.

Reviewer #2: Yes

**********

5. Is the manuscript presented in an intelligible fashion and written in standard English?

PLOS ONE does not copyedit accepted manuscripts, so the language in submitted articles must be clear, correct, and unambiguous. Any typographical or grammatical errors should be corrected at revision, so please note any specific errors here.

Reviewer #2: Yes

**********

6. Review Comments to the Author

Please use the space provided to explain your answers to the questions above. You may also include additional comments for the author, including concerns about dual publication, research ethics, or publication ethics. (Please upload your review as an attachment if it exceeds 20,000 characters)

Reviewer #2: The authors have addressed my comments thoroughly and in good faith. The description of the hospital setting is now substantially more informative, and the breakdown of participants by clinical unit improves the transparency of the sample characterisation. The discussion of younger and less experienced nurses represents a meaningful addition that contextualises the descriptive findings within a broader workforce perspective. Most importantly, the revised discussion now explicitly acknowledges the tension between structural determinants of burnout and individually-oriented recommendations — a critical point that significantly strengthens the manuscript's intellectual honesty and public health relevance.

One remaining suggestion for the final version: the limitations section acknowledges the omission of salary-related variables but does not discuss the potential direction of this bias. Given that compensation in South Korean public hospitals typically differs from the private sector, unmeasured financial dissatisfaction could confound the observed association between workload and burnout. A brief sentence addressing this would further strengthen the limitations paragraph.

**********

7. PLOS authors have the option to publish the peer review history of their article (what does this mean?). If published, this will include your full peer review and any attached files.

If you choose “no”, your identity will remain anonymous but your review may still be made public.

**Do you want your identity to be public for this peer review?**  For information about this choice, including consent withdrawal, please see our Privacy Policy.

Reviewer #2: **Yes:**  Xacobe Fernández-García

**********

# Editor report: Acceptance letter

**DOI:** 10.1371/journal.pone.0354729.r004

**Authors:** Rogis Baker

PONE-D-26-11716R1

PLOS One

Dear Dr. Jin,

I'm pleased to inform you that your manuscript has been deemed suitable for publication in PLOS One. Congratulations! Your manuscript is now being handed over to our production team.

At this stage, our production department will prepare your paper for publication. This includes ensuring the following:

* All references, tables, and figures are properly cited

* All relevant supporting information is included in the manuscript submission,

* There are no issues that prevent the paper from being properly typeset

You will receive further instructions from the production team, including instructions on how to review your proof when it is ready. Please keep in mind that we are working through a large volume of accepted articles, so please give us a few days to review your paper and let you know the next and final steps.

Lastly, if your institution or institutions have a press office, please let them know about your upcoming paper now to help maximize its impact. If they'll be preparing press materials, please inform our press team within the next 48 hours. Your manuscript will remain under strict press embargo until 2 pm Eastern Time on the date of publication. For more information, please contact onepress@plos.org.

You will receive an invoice from PLOS for your publication fee after your manuscript has reached the completed accept phase. If you receive an email requesting payment before acceptance or for any other service, this may be a phishing scheme. Learn how to identify phishing emails and protect your accounts at https://explore.plos.org/phishing.

If we can help with anything else, please email us at customercare@plos.org.

Thank you for submitting your work to PLOS ONE and supporting open access.

Kind regards,

PLOS ONE Editorial Office Staff

on behalf of

Dr. Rogis Baker

Academic Editor

PLOS One
